# Autoimmune glial fibrillary acidic protein astrocytopathy mimicking acute disseminated encephalomyelitis: A case report

**DOI:** 10.1097/MD.0000000000026448

**Published:** 2021-06-25

**Authors:** Jiao Li, Chentao Wang, Yulan Cao, Jijun Shi, Huihui Liu, Meili Zhou, Chunfeng Liu, Weidong Hu

**Affiliations:** Department of Neurology, The Second Affiliated Hospital of Soochow University, Suzhou, China.

**Keywords:** acute disseminated encephalomyelitis, autoimmune glial fibrillary acidic protein astrocytopathy, glial fibrillary acidic protein-IgG, peripheral nervous system

## Abstract

**Introduction:**

Autoimmune glial fibrillary acidic protein (GFAP) astrocytopathy is an increasingly recognized type of steroid-responsive autoimmune disease of the nervous system. Defined in 2016, it is associated with the presence of anti-GFAP immunoglobulinG in the serum or cerebrospinal fluid (CSF) of affected patients.

**Patient characteristics:**

Herein, we report a case of acute neurological symptoms, including headache, fever, confusion, and paralysis of the lower extremities. CSF analysis revealed lymphocytic pleocytosis and elevated protein levels, indicating acute disseminated encephalomyelitis, and the patient was given immunotherapy. Cranial magnetic resonance imaging showed multifocal T2/fluid-attenuated inversion recovery hyperintense signal changes in the periventricular white matter, and electromyography testing showed changes consistent with severe sensorimotor neuropathy, indicating the involvement of the brain and peripheral nerves.

**Diagnoses:**

Finally, a diagnosis of autoimmune GFAP astrocytopathy was confirmed due to the presence of GFAP-immunoglobulinG in the patient's CSF.

**Interventions:**

The patient was treated with one course of intravenous immunoglobulin therapy, then followed with intravenous methylprednisolone (1.0 g/d for 3 days) and oral prednisolone.

**Outcomes:**

At 1 week after intravenous immunoglobulin therapy, his level of consciousness improved. However, flaccid paralysis persisted without substantial improvement.

**Conclusion:**

In conclusion, the provision of an accurate early diagnosis and appropriate treatment are crucial for improving the prognosis of patients with autoimmune GFAP astrocytopathy. Further, this case highlights the importance of recognizing the role of peripheral nerve involvement in GFAP autoimmunity.

## 1 Introduction

Autoimmune glial fibrillary acidic protein (GFAP) astrocytopathy is increasingly recognized as a form of a nervous system steroid-responsive autoimmune disease. Defined in 2016, it is associated with the presence of anti-GFAP immunoglobulinG (IgG) in a patient's serum or cerebrospinal fluid (CSF).^[1,2]^ Most frequently GFAP astrocytopathy clinically resembles acute-onset meningoencephalitis with or without spinal cord involvement, and presents as headache, subacute encephalopathy, seizures, psychosis, cerebellar ataxia, optic neuritis, and inflammatory myelitis.^[3]^ In addition, autoimmune GFAP astrocytopathy sometimes is associated with neurological and systemic autoimmunity and autoantibodies.^[4]^ Most cases of the disease involve the presence of inflammatory CSF, and its characteristic radiological hallmark is brain linear perivascular radial gadolinium enhancement on magnetic resonance imaging (MRI).^[2]^

Herein, we report a case of autoimmune GFAP astrocytopathy whose clinical manifestation, cerebrospinal fluid (CSF) results and imaging highly mimic acute disseminated encephalomyelitis. Subsequently, the expression of GFAP-IgG in the CSF lead to a final autoimmune GFAP astrocytopathy diagnosis.

## 2 Case report

A 56-year-old Chinese man was admitted to the hospital with headache, a fever of 38 to 39 °C for 4 days, confusion, and paralysis of the lower extremities. His medical history included hypertension and untreated psoriasis. His family history was unremarkable. A neurological examination revealed that the patient had a reduced alertness level, neck stiffness, flaccid paralysis of limbs with diffuse areflexia, and no spontaneous limb movements. Brain computed tomography revealed no edema, space-occupying lesions, intracranial hemorrhage, or collections. Blood tests showed hyponatremia (Na, 127.9 mmol/L) and hypochloremia (Cl, 91.1 mmol/L), while routine laboratory studies including routine blood, C-reactive protein, coagulation tests, and liver and renal function tests were all unremarkable. He was admitted to the neurology ward with a provisional diagnosis of central nervous system infection. His condition deteriorated rapidly, and he was intubated due to coma and respiratory failure.

Initial cerebrospinal fluid (CSF) analysis revealed lymphocytic pleocytosis, with a white blood cell (WBC) count of 392 × 10^6^/L (normal: 0–8 × 10^6^/L) and 95% lymphocytes. The CSF examination also revealed elevated protein, glucose, chloride, and adenosine deaminase (ADA) levels of 1773 mg/L (normal: <450 mg/L), 3.39 mmol/L (normal: 2.50–4.50 mmol/L), 110.0 mmol/L (normal: 120–132 mmol/L), and 4 U/L (normal: 0–25 U/L), respectively. Metagenomic next-generation sequencing of viral and bacterial genomes from the CSF was performed, which was positive exclusively for Epstein-Barr (EB) virus DNA. In addition, a serum viral assay revealed the presence of Epstein-Barr virus (EBV) DNA and EBV-capsid antigen (CA)-IgG, but was negative for EBV-CA-IgM, EBV-CA-IgA, and EBV-early antigen (EA)-IgG. Therefore, the patient was initially diagnosed with infectious meningoencephalitis and was treated with 300 mg intravenous ganciclovir twice daily and 4.0 ceftriaxone sodium daily.

Further autoimmune encephalitis panel testing for antibodies to anti-aquaporin 4 (AQP4), anti-myelin oligodendrocyte glycoprotein (MOG), anti-N-methyl-D-aspartate receptor (NMDAR), anti-LGI1, and anti-GABA_B_R in the patient's CSF and serum were negative. Moreover, line blots for anti-Hu, anti-Yo, anti-Ri, anti-amphiphysin, anti-CV2, anti-Ma1, and anti-Ma2 were also negative. No anti-GFAP test was performed at that time. Electroencephalography (EEG) findings were mostly normal, and no epileptiform activity was observed. Electromyography testing (EMG) showed that compound muscle action potentials (CMAPs) were absent in the tibial and common peroneal nerves of both lower extremities, and bilateral sural sensory nerve conduction velocities (SNCVs) were decreased, indicating severe, predominantly axonal, sensorimotor neuropathy.

Acyclovir was continued, and the patient was empirically treated for infectious meningoencephalitis with ceftriaxone and meropenem. However, his neurological condition progressively worsened. A lumbar puncture was repeated on day 14. At that time, the patient had a markedly elevated total protein concentration in the CSF of 2772 mg/L, along with a WBC concentration of 195 × 10^6^cells/L (95% lymphocytes), a glucose level of 4.11 mmol/L, and ADA concentration of 6 U/L. These findings were inconsistent those that are expected due to typical bacterial and viral forms of meningoencephalitis. Thus, a demyelinating condition, such as acute disseminated encephalomyelitis (ADEM), which could explain the patient's clinical symptoms was suggested. Therefore, the patient was treated with one course of intravenous immunoglobulin (IVIG) therapy (400 mg/kg/d for 5 consecutive days). His clinical condition gradually improved, and he was extubated.

**Figure 1:**
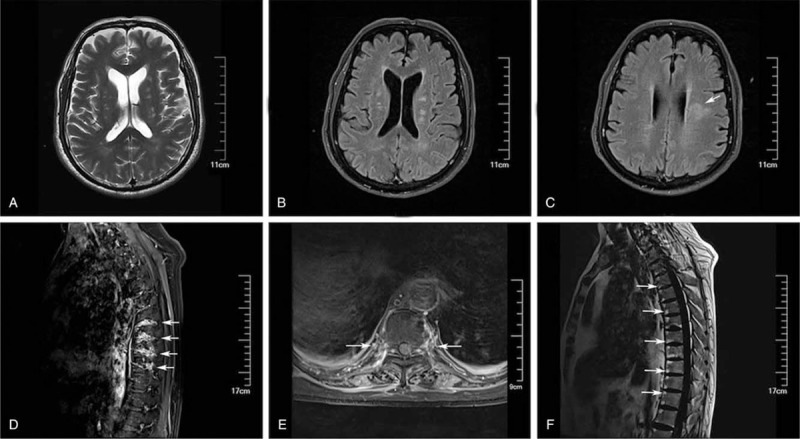
An MRI series of the brain and spine of the patient. Figure panels depict the following findings: (A) a brain MRI revealed T2 hyperintense lesions in the brainstem; (B) a brain MRI revealed multifocal T2 hyperintense lesions within cerebral white matter; (C) a corresponding fluid attenuated inversion recovery (FLAIR) sequence shows hyperintense signals in cerebral white matter; (D) a sagittal T1 post-contrast fat saturated Dixon sequence of the spine revealed enhancement of thoracic nerve roots; (E) a corresponding axial MRI of thoracic spine; and (F) a spinal T_1_-weighted MRI shows multiple spondylitis of anterior corners. MRI = magnetic resonance imaging.

Initially, cranial MRI showed a multifocal T2/fluid-attenuated inversion recovery hyperintense signal change in the periventricular white matter involving the following structures: bilateral centrum semiovale, corona radiata, and brainstem (Fig. 1A–C). In addition, spinal MRI showed enhancement of the thoracic nerve roots and lumbar paraspinal muscles (Fig. 1D and E). In addition, spinal MRI confirmed multiple spondylitis of the anterior and posterior vertebral corners, suggesting the possibility of psoriatic spondylitis with axial involvement (Fig. 1F).

Using cell-based assays, we detected GFAP-IgG in the CSF of the patient, but not in his serum. Taken together, these findings allowed us to make definite diagnosis of autoimmune GFAP astrocytopathy. Therefore, he was treated with intravenous 1.0 g/d methylprednisolone pulse therapy for 3 consecutive days, followed by an initial 60 mg/d oral prednisolone dose before the drug was tapered gradually. After 1 month of treatment, we performed another CSF examination, which revealed a normal WBC count and decreased protein levels (1171 mg/dL, normal: <450 mg/L). However, the patient's flaccid paralysis persisted without substantial improvement. At a follow-up appointment 6 months later, the patient had sequelae of biparaplegia.

## 3 Discussion

There are no uniform diagnostic criteria for autoimmune GFAP astrocytopathy.^[5]^ Currently, its diagnosis is based on the presence of GFAP antibodies in a patient's CSF or serum. In the present study, we made a final diagnosis of autoimmune GFAP astrocytopathy in a patient with psoriatic spondylitis. The diagnosis was confirmed by the acute onset of meningoencephalitis and the detection of GFAP antibodies in the patient's CSF.

The relationship between virus infection and GFAP astrocytopathy is unclear.^[5]^ This patient had symptoms that included headache and fever in the early stage, and developed disturbances of consciousness. Because the CSF next-generation sequencing screen was positive for EBV, this case observationally links EBV infection and autoimmune GFAP astrocytopathy. EBV in the CSF should not always be interpreted as a cause of neurological manifestations.^[6]^ EBV has also been linked to the development of autoimmunity. While few studies have previously reported an association between autoimmune GFAP astrocytopathy and herpes virus infection,^[7]^ infectious triggers have been proposed as a potential mechanism that underlies autoimmune encephalitis.^[8]^ Therefore, we speculated that the following different pathophysiologies were responsible: EBV infection may damage the blood-brain barrier and invade the central nervous system; EBV molecular mimicry or latently infected B cells may contribute to an immune-mediated autoimmune response post-infection.

As noted for classic autoimmune neurologic diseases, it has been previously reported that patients with autoimmune GFAP astrocytopathy often have coexisting autoimmune disease, suggesting antibody-mediated immune susceptibility and immune-mediated disease mechanisms.^[4,9]^ Interestingly, our patient had concomitant psoriatic spondylitis with axial involvement, which was confirmed by imaging, and he was HLA-B27 antigen positive. Psoriatic arthritis was originally considered to be a Th1-mediated disease.^[10]^ However, GFAP was reported to be mediated by GFAP peptide-specific cytotoxic CD8+ T cells in a transgenic mouse model of autoimmune GFAP meningoencephalitis.^[11]^ Therefore, further investigation is needed to determine whether shared immunoinflammatory pathways trigger the coexistence of both diseases.

Our patient presented with a clinical history, symptoms, physical examination, and CSF profile suggestive of ADEM. Although ADEM usually affects children and young adults, some cases have been reported in older adults.^[12]^ A diagnosis of probable ADEM was made, followed by immunotherapy. Finally, an autoimmune GFAP astrocytopathy diagnosis was confirmed by the presence of GFAP-IgG in the CSF. Notably, this patient displayed lower extremity weakness, consistent with electrophysiological evidence of axonal sensorimotor neuropathy, which caused flaccid paralysis that was unresponsive to immunotherapy. Given the reported association between GFAP IgG and peripheral polyneuropathy,^[13,14]^ it is interesting to speculate that the peripheral nervous system may be involved in autoimmune GFAP astrocytopathy. One limitation of this study is that pathologic tissue confirmation of peripheral nerve involvement was not obtained.

Laboratory tests showed that the patient displayed hyponatremia and hypochloremia on admission, and chloride levels in the CSF decreased slightly. This is consistent a prior report that showed that over half of patients present with hyponatremia.^[3]^ The exact cause of hyponatremia remains unclear. In the case described here, the patient behaved clinically and biochemically as if he had excessive levels of anti-diuretic hormone, low serum osmolality, and inappropriately high urine osmolality.

The treatment of GFAP astrocytopathy in the acute stage includes high-dose corticosteroids, IVIG, and plasma exchange. Most patients respond well to steroid therapy although some patients had a poor response to treatment or even died, and some patients were left with different degrees of functional disability.^[15]^ Although this patient had a poor corticosteroid response and flaccid paralysis persisted even after corticosteroids therapy, it seems reasonable to postulate that administration of IVIG may have played a causal rather than a coincidental role in the transformation from confusion to wakefulness.

In conclusion, accurate early diagnosis and appropriate treatment are crucial for improving the prognosis of patients with autoimmune GFAP astrocytopathy. We suggest that GFAP-IgG detection should be included in the comprehensive evaluation of patients with meningoencephalitis.

## Acknowledgments

The authors would like to express their sincere gratitude to the patient for their understanding and participation in this study.

## Author contributions

**Conceptualization:** Huihui Liu, Meili Zhou.

**Data curation:** Yulan Cao, Jijun Shi.

**Investigation:** Jiao Li, Chentao Wang.

**Supervision:** Chunfeng Liu, Weidong Hu.

**Writing – original draft:** Jiao Li.

**Writing – review & editing:** Weidong Hu.
